# A Milky Mystery: Chylothorax Unmasking Non-Hodgkin Lymphoma

**DOI:** 10.7759/cureus.93559

**Published:** 2025-09-30

**Authors:** Shivendra Tangutoori, Dedeepya Gullapalli, Sai Subramanyam Kommineni, Mythili kanthi Gudipati, Jayaramakrishna Depa, Maneesh Gaddam, Subramanya Shyam Ganti

**Affiliations:** 1 Internal Medicine, Appalachian Regional Healthcare, Harlan, USA; 2 Nephrology, Appalachian Regional Healthcare, Harlan, USA; 3 Pulmonary and Critical Care, Hazard ARH Regional Medical Center, Hazard, USA; 4 Internal Medicine/Pulmonary Critical Care, Appalachian Regional Healthcare, Harlan, USA

**Keywords:** malignant chylothorax, milky pleural fluid, non-hodgkin lymphoma, non-traumatic chylothorax, oncology case report

## Abstract

Chylothorax is an uncommon cause of pleural effusion that presents diagnostic and therapeutic challenges. We report the case of a 71-year-old woman who presented with shortness of breath and abdominal distension for one week. Thoracic imaging revealed a large left-sided pleural effusion. A thoracentesis drained 1 liter of turbid, milky fluid, and pleural fluid analysis confirmed an exudative effusion with a triglyceride level of 1199 mg/dL. Cytology identified CD markers (CD10, CD19, CD20) consistent with stage IV follicular lymphoma. The patient was treated with six cycles of R-CHOP (rituximab, vincristine, cyclophosphamide, and prednisone) chemotherapy and required a pleurX catheter for recurrent effusions, achieving partial symptomatic control. This case highlights malignant chylothorax as a rare clinical presentation of non-Hodgkin lymphoma and underscores the importance of a multidisciplinary approach in diagnosis and management. To our knowledge, this represents a rare instance of follicular lymphoma initially manifesting as chylothorax.

## Introduction

Chyle is a lymphatic fluid rich in triglycerides, fat-soluble vitamins, and lymphocytes, derived from digested fats and transported via the thoracic duct into the venous circulation [1]. Chylothorax, the accumulation of chyle within the pleural cavity, is diagnosed when pleural fluid triglyceride levels exceed 110 mg/dL or chylomicrons are detected, while cholesterol levels are typically below 200 mg/dL [2]. 

It is uncommon, accounting for approximately 3% of all pleural effusions. Causes are broadly divided into traumatic (e.g., postoperative thoracic duct injury) and non-traumatic, with malignancy, especially lymphoma, accounting for the majority of non-traumatic cases [3]. In fact, lymphomas are responsible for up to 70-75% of malignant chylothorax, with non-Hodgkin lymphoma being the predominant cause [4]. Although uncommon, chylothorax carries significant morbidity and mortality due to nutritional depletion, metabolic derangements, and immune compromise, with malignant chylothorax being particularly associated with poor outcomes [5].

This case is unique because follicular lymphoma presenting initially with malignant chylothorax is rare, with only a limited number of cases described in recent literature [6,7]. Despite therapeutic advances, malignant chylothorax remains associated with high morbidity and variable survival, underscoring the clinical relevance of reporting such cases. We present one such rare case which presented with chylothorax that was later diagnosed with follicular lymphoma.

## Case presentation

**Figure 1:**
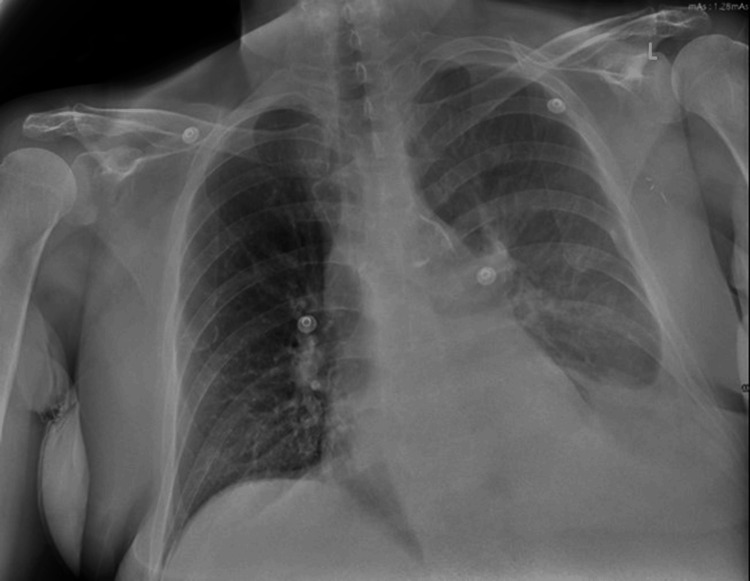
Chest X-ray showing left lower lobe opacity and blunting of the costophrenic angle consistent with pleural effusion

A 71-year-old woman with a past medical history of left-sided invasive ductal carcinoma, stage IIA, status post lumpectomy four years ago (with no evidence of recurrence and declined adjuvant chemo-radiation), presented to the hospital with shortness of breath and abdominal distention for one week. She denied fevers, chills, night sweats, or hemoptysis. On physical exam, she was mildly tachypneic, with decreased breath sounds over the left lung bases and 1+ pitting edema in bilateral lower extremities. Her vital signs were stable, and she maintained an oxygen saturation of 99% on room air. Chest radiography (Figure 1) showed a left lower lobe opacity with blunting of the left costophrenic angle.

**Figure 2:**
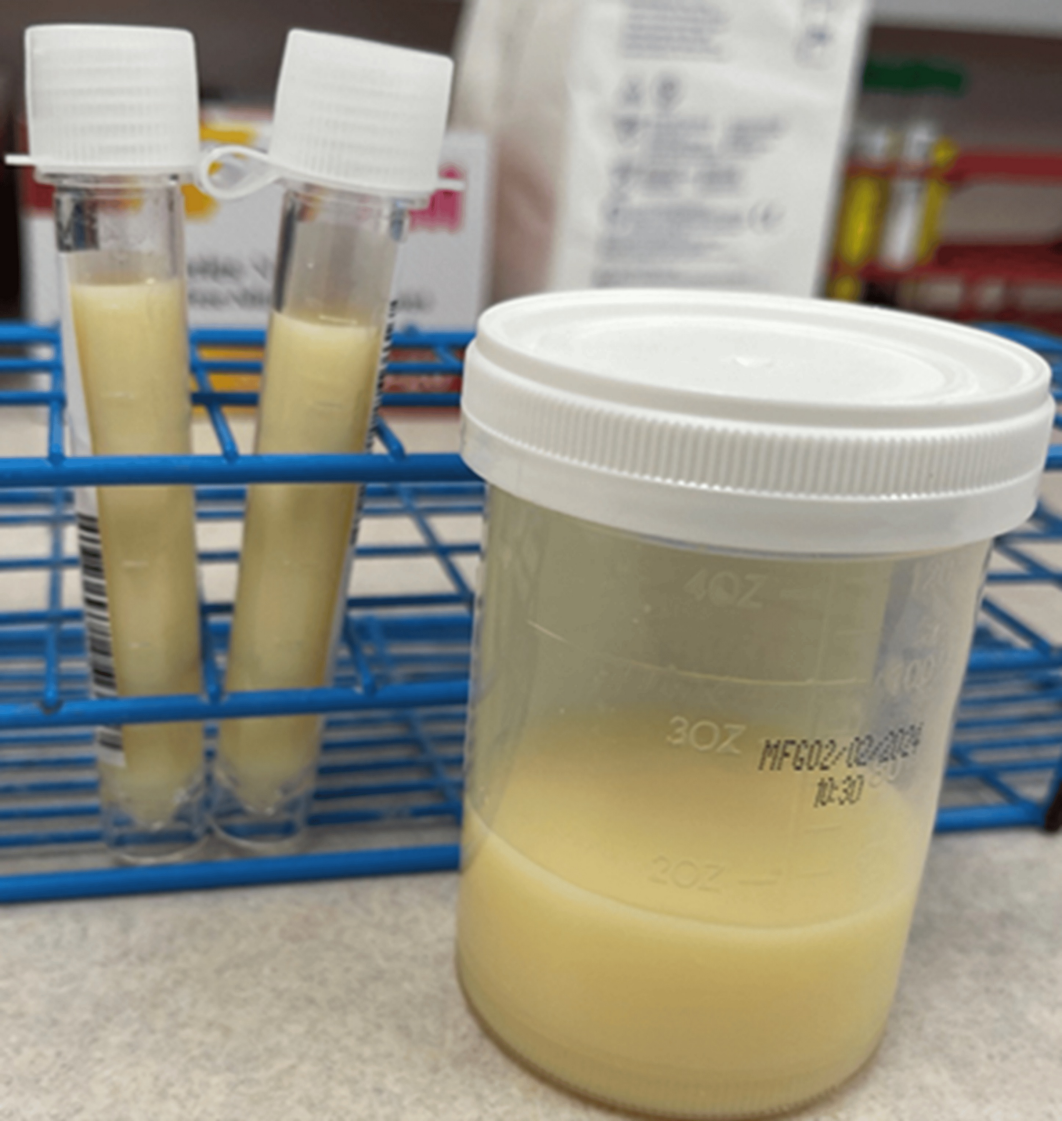
Container showing milky, turbid pleural fluid obtained via thoracentesis, consistent with chylothorax Image Credits: Subramanya Shyam Ganti (pic taken after the thoracentesis, before sending to lab for analysis)

A computerized tomography (CT) scan of the abdomen and pelvis revealed extensive bulky retroperitoneal and mesenteric adenopathy, with a large mass measuring 15 x 9.8 cm, highly suspicious for malignancy. Additionally, the scan showed new-onset moderate ascites, incompletely visualized left-sided breast masses, and moderate-to-large left and trace right pleural effusions. Ultrasound imaging confirmed the presence of ascites, but the volume was insufficient for a safe paracentesis. A large fluid pocket was identified in the left pleural cavity, and a diagnostic and therapeutic thoracentesis was performed. The pleural fluid appeared turbid and milky (Figure 2) upon aspiration, and approximately one liter of pleural fluid was drained without any complications. Pleural fluid analysis confirmed an exudative chylous effusion (Table 1), with serum LDH of 393 IU/L and serum protein of 4.9mg/dl.

**Table 1:** Pleural fluid analysis results with the normal reference range IU: International Units; L: liter; dL: deciliter; mg: milligram; g: gram

Fluid Characteristic	Patient’s Pleural Fluid Value	Normal Reference Range
Fluid pH	7.5	7.40-7.45
Fluid glucose (mg/dL)	89	30-60
Fluid total protein (g/dL)	3.0	<3gm/dl; <50% of serum protein (transudative); >50% exudative
Fluid albumin (mg/dL)	2.3	No standard cutoff; usually compared to serum
Fluid LDH (IU/L)	262	135-225; also compared with serum LDH to determine the nature
Fluid amylase (IU/L)	18	<100
Fluid lipase (IU/L)	11	<160
Fluid cholesterol (mg/dL)	97	<200
Fluid triglycerides (mg/dL)	1199	<110

The pleural fluid cytology revealed lymphocytosis with small mature lymphocytes. Flow cytometry demonstrated a clonal B-cell population expressing CD10, CD19, CD20, and surface lambda light chain restriction, consistent with follicular lymphoma. Peritoneal fluid analysis showed only chronic inflammatory cells. A retroperitoneal mass biopsy later confirmed follicular lymphoma with BCL2 rearrangement and germinal center origin.

The patient was started on aggressive chemotherapy with six cycles of R-CHOP (rituximab, vincristine, cyclophosphamide, and prednisone). She experienced recurrent effusions during treatment, requiring an indwelling pleurX catheter with daily output of 500-600 mL. Following completion of chemotherapy, she demonstrated a partial response with reduced adenopathy and improved respiratory symptoms, though the effusion persisted intermittently. She continues oncology follow-up.

## Discussion

Chyle contains chylomicrons that transport triglycerides from the intestines to the lymphatic system, eventually draining into the venous circulation via the thoracic duct [8]. This extravasation of chyle into the pleural space due to obstruction or injury to the thoracic duct or transdiaphragmatic flow from the peritoneal cavity leads to chylothorax [9,10]. In our case, the malignant chylothorax was secondary to follicular lymphoma, causing lymphatic obstruction. Less than half of the chylothoraces are milky in appearance [9]. One other cause for milky-appearing pleural fluid is pseudo-chylothorax, which is a cholesterol-rich (>200 mg/dl) fluid with triglycerides <110 mg/dl and a pleural cholesterol to triglyceride level ratio greater than 1, with cholesterol crystals in the microscopy [11]. It is associated with chronic inflammation [2]. In cirrhotic chylothorax or chylous ascites, a subdiaphragmatic lymphatic leak may result in transdiaphragmatic flow of chyle from the peritoneal cavity into the pleural space [12]. This highlights the importance of imaging and diagnostic confirmation to distinguish malignant chylothorax from other causes.

Chylothorax occurs unilaterally in 84% of cases, with 50-60% of all cases occurring on the right side [2,9]. The clinical presentation of chylothorax varies depending on the rate of chyle accumulation in the pleural space. The most common symptoms include cough and shortness of breath, while malignancy-related chylothorax may present with systemic symptoms such as night sweats, weight loss, and asthenia [5]. Fever and pleuritic pain are rarely seen, as chyle itself does not irritate the pleural surface [13]. Pleural fluid analysis in chylothorax can be transudative or exudative. Traumatic causes are subdivided into surgical and non-surgical etiologies. The two major surgical causes include esophagectomy and repair of congenital heart disease [14]. Non-traumatic causes are the majority, including malignancies, specifically lymphomas; lymphatic disorders like lymphangioleiomyomatosis, pulmonary lymphangiectasia, and lymphangiomas; other etiologies include chylous ascites, sarcoidosis, and infections like Mycobacterium tuberculosis, which is the most common, including other infections like hepatitis A and paracoccidioidomycosis [15,16].

**Figure 3:**
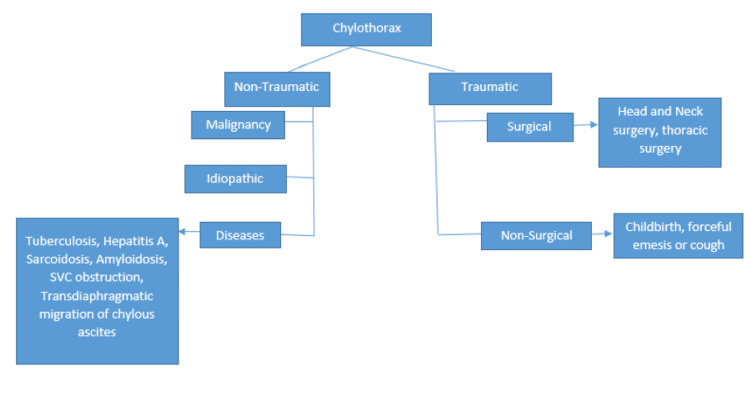
Flowchart showing the causes and classification of chylothorax into traumatic and non-traumatic causes

Diagnosing non-traumatic chylothorax involves two main steps: (i) confirming the diagnosis of chylothorax and (ii) determining the underlying etiology [17].

The gross appearance of pleural fluid is neither sensitive nor specific for diagnosing chylothorax. The variation in fluid color can be attributed to differences in dietary lipid intake, with lower triglyceride consumption resulting in less turbid effusions [5]. Sudan III or IV staining can help identify chylomicrons in pleural fluid but lacks specificity for definitive diagnosis [18]. The most reliable diagnostic test for chylothorax is a pleural fluid triglyceride level >110 mg/dL with a cholesterol level <200 mg/dL, a consistent finding in our patient. However, if the triglycerides are less than 110 mg/dL but the clinical history or pleural effusion appearance is consistent with a possible etiology of chylothorax, then the pleural chylomicron study can be performed to confirm the diagnosis. Transudative chylothorax is an uncommon condition. A retrospective study by Agrawal et al. [9] identified cases associated with heart failure, superior vena cava obstruction, and pulmonary hypertension. Additionally, a case report by Do et al. [19] described transudative chylothorax as a complication of recurrent chylous ascites. Once the diagnosis is confirmed, pleural fluid characteristics might help us establish the etiology. If it is a lymphocyte-predominant, protein-discordant, exudative effusion, then it is an isolated chylothorax, but if it is a non-lymphocyte-predominant, protein-discordant, exudative effusion, an alternate cause should be identified [9].

Other modalities in finding out the cause include imaging studies such as chest radiography, thoracic ultrasound, and CT of the thorax and abdomen, or lymphangiography. CT imaging, particularly in cases of suspected malignancy-related chylothorax, is useful for detecting compressive mediastinal or abdominal lymphadenopathy or malignant lesions compressing the thoracic duct, as in our case [7]. Lymphangiography, or lymphography, includes injecting a poppyseed-based oil into the foot or ankle lymphatic vessel, and then the thoracic duct is evaluated in a conventional radiography or CT, which helps in identifying the lymphatic leak, chylothorax, and chylous ascites [20]. The treatment of chylothorax primarily involves addressing the underlying cause. Based on the chest tube output, treatment is considered. If the output is less, it is managed conservatively with drainage, dietary modification (medium-chain triglyceride diet or fat restriction <10 g/day), and somatostatin analogues. Somatostatin analogues bind to the somatostatin receptors and reduce the chyle production, lymph flow, and fat absorption [21]. Patients with chylothorax are advised to have high high-protein and low-fat diet (<10 g/day) [22]. If the chest tube output is high, i.e., 1.1 L in 24 hours or 1 L/day for more than five days or 2 L/day in two days of optimal conservative therapy, then surgical intervention can be considered. Surgical interventions include thoracic duct ligation, thoracic duct embolization or disruption, and pleurodesis [23]. In lymphoma-associated chylothorax, systemic chemotherapy remains the cornerstone, with pleural catheters offering palliation when effusions recur. In malignant chylothorax, prognosis is generally poor, particularly when associated with advanced lymphoma (study done by Porcel et al. [24]). In our patient, systemic chemotherapy with R-CHOP was initiated, leading to partial control of effusions. Recurrent effusions necessitated placement of a PleurX catheter, which allowed individualized management [25]. Compared with other reported cases, follicular lymphoma presenting initially with chylothorax is rare, and response to standard therapy may vary [26].

**Figure 4:**
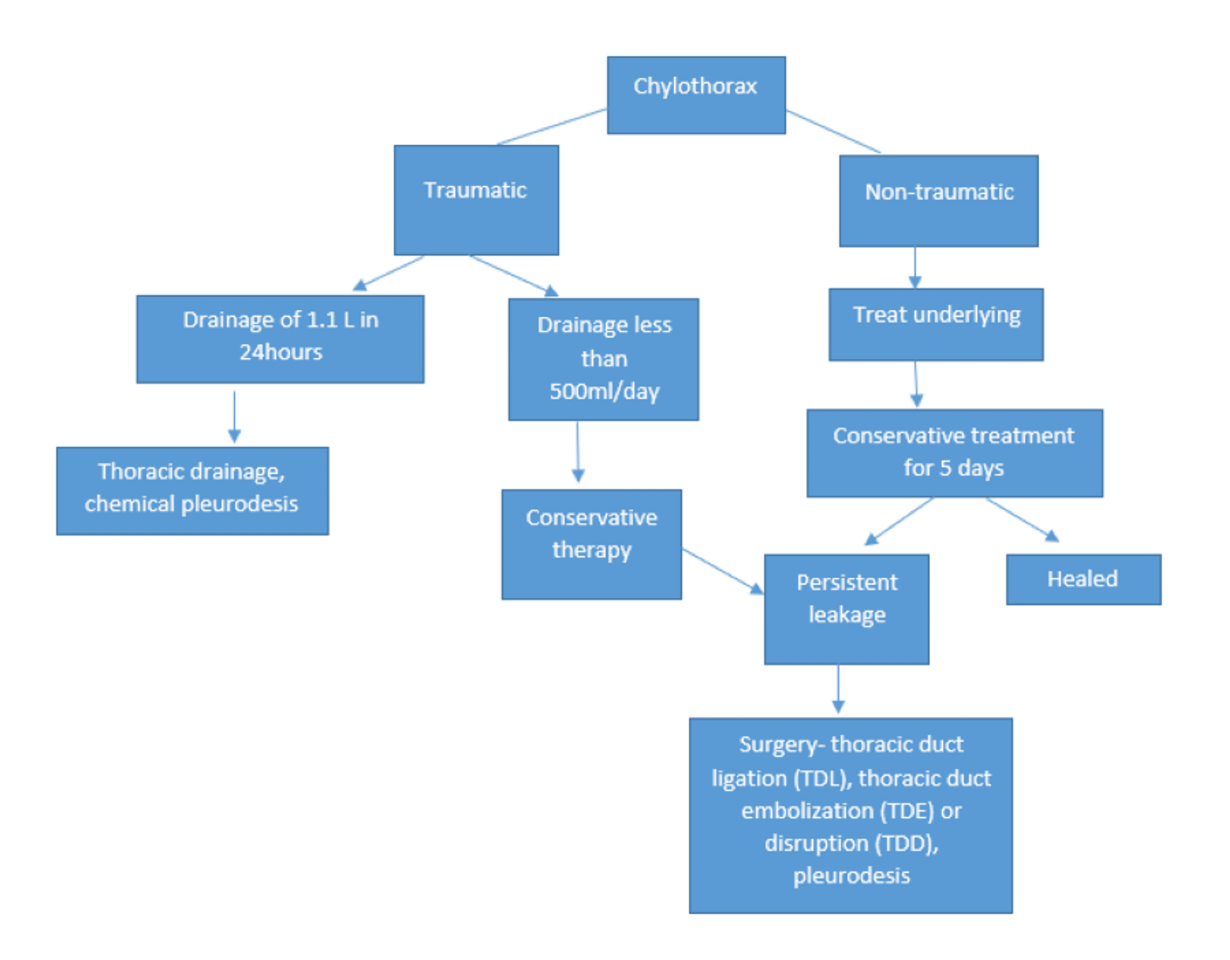
Flowchart showing management of chylothorax

## Conclusions

Non-traumatic chylothorax occurs due to disruption of lymphatic flow without trauma. Diagnosis relies on pleural fluid triglycerides >110 mg/dL with cholesterol <200 mg/dL or detection of chylomicrons. Once the diagnosis is confirmed, understanding the underlying pleural fluid characteristics/pattern helps us identify the underlying cause is important for early management and prognostic purposes. This case underscores a key teaching point: malignant chylothorax can be the initial manifestation of follicular lymphoma, a rare but clinically significant presentation. It also highlights the diagnostic challenge in patients with prior malignancy, such as breast carcinoma, where differentiating between metastatic disease and a new hematologic malignancy is crucial. Management requires a multidisciplinary approach addressing both the effusion and the underlying malignancy. Future studies are needed to evaluate outcomes in lymphoma-associated chylothorax in the era of modern therapies.

## References

[1] Sassoon CS, Light RW (1985). Chylothorax and pseudochylothorax. Clin Chest Med.

[2] Huggins JT (2010). Chylothorax and cholesterol pleural effusion. Semin Respir Crit Care Med.

[3] Rudrappa M, Paul M (2025). Chylothorax. StatPearls [Internet].

[4] Rajdev K, Avula A, Sharma D, Mansour W, Agarwal S, Siddiqui AH, Chalhoub M (2018). A case of transudative chylothorax: a diagnostic dilemma. Cureus.

[5] Pillay TG, Singh B (2016). A review of traumatic chylothorax. Injury.

[6] Bhatnagar M, Fisher A, Ramsaroop S, Carter A, Pippard B (2024). Chylothorax: pathophysiology, diagnosis, and management-a comprehensive review. J Thorac Dis.

[7] Kim GE, Khaled Y, Mahmoud S, Rehman AU (2024). Chylothorax as initial presentation of follicular lymphoma: a case report and literature search. Case Rep Hematol.

[8] Nadolski G (2016). Nontraumatic chylothorax: diagnostic algorithm and treatment options. Tech Vasc Interv Radiol.

[9] Agrawal V, Doelken P, Sahn SA (2008). Pleural fluid analysis in chylous pleural effusion. Chest.

[10] Skouras V, Kalomenidis I (2010). Chylothorax: diagnostic approach. Curr Opin Pulm Med.

[11] Agrawal V, Sahn SA (2008). Lipid pleural effusions. Am J Med Sci.

[12] Akbar A, Hendrickson T, Vangara A, Marlowe S, Hussain A, Ganti SS (2023). Hepatic chylothorax: an uncommon pleural effusion. J Investig Med High Impact Case Rep.

[13] Romero S (2000). Nontraumatic chylothorax. Curr Opin Pulm Med.

[14] Doerr CH, Allen MS, Nichols FC 3rd, Ryu JH (2005). Etiology of chylothorax in 203 patients. Mayo Clin Proc.

[15] Mehta K, Shinde S, Rego S, Shet A (2015). Hepatitis A associated with chylothorax: an uncommon presentation of a common infection. J Trop Pediatr.

[16] Wright RS, Jean M, Rochelle K, Fisk D (2011). Chylothorax caused by Paragonimus westermani in a native Californian. Chest.

[17] de Beer HG, Mol MJ, Janssen JP (2000). Chylothorax. Neth J Med.

[18] Staats B, Ellefson R, Budahn L (1980). The lipoprotein profile of chylous and nonchylous pleural effusions. Mayo Clin Proc.

[19] Do TV, Cozza J, Ganti S, Depa J (2021). Recurrent chylous ascites leading to transudative chylothorax due to bi-ventricular heart failure. J Investig Med High Impact Case Rep.

[20] Guermazi A, Brice P, Hennequin C, Sarfati E (2003). Lymphography: an old technique retains its usefulness. Radiographics.

[21] Al-Zubairy SA, Al-Jazairi AS (2003). Octreotide as a therapeutic option for management of chylothorax. Ann Pharmacother.

[22] Sriram K, Meguid RA, Meguid MM (2016). Nutritional support in adults with chyle leaks. Nutrition.

[23] Selle JG, Snyder WH 3rd, Schreiber JT (1973). Chylothorax: indications for surgery. Ann Surg.

[24] Porcel JM, Bielsa S, Civit C (2023). Clinical characteristics of chylothorax: results from the International Collaborative Effusion database. ERJ Open Res.

[25] Milsom JW, Kron IL, Rheuban KS, Rodgers BM (1985). Chylothorax: an assessment of current surgical management. J Thorac Cardiovasc Surg.

[26] Maral S, Albayrak M, Afacan Öztürk B (2021). Case report: Follicular lymphoma presented with chylothorax. Hematol Transfus Cell Ther.

